# What is public health? a scoping review

**DOI:** 10.1186/s13690-023-01091-6

**Published:** 2023-05-10

**Authors:** Razieh Azari, Bettina Borisch

**Affiliations:** 1 Institute of Global Health, Faculty of Medicine, University of Geneva, Geneva, 1202 Switzerland; 2 World Federation of Public Health Associations, WFPHA, Geneva, Switzerland

**Keywords:** Public health, Public health definition, Public health meaning, Scoping review

## Abstract

**Background:**

during the last hundred years, several major public health issues have already afflicted humanity. Most frequently cited definitions of public health have stemmed from long-standing definitions, which raises several concerns including whether these definitions can respond to today’s public health challenges. The present study aimed to identify and review available public health definitions in the first place.

**Methods:**

in this scoping review, we undertook an electronic search in four databases (PubMed, Web of Science, Embase, and EBSCOhost) from inception until June 06, 2022, and a grey literature search in Google Scholar. Moreover, reference lists of publications included in the scoping review were screened manually for additional relevant publications. All types of scientific publications, in English, that focused on the definition of public health and provided an original definition were included. Year, type, disciplinary fields of publications, objectives of publications, and public health definitions were extracted.

**Results:**

5651 publications were identified through the scoping search, of which five were subjected to full-text review. Of these publications, two were included. An additional nine publications were identified through the manual screening. A total 11 of publications were included in the scoping review. Of the 11 definitions included in this review, the latest original definitions date back to about two decades ago.

**Conclusions:**

there is a noticeable lack of updated definitions of public health. Considering our findings and the ever-changing nature of public health issues, there is an urgent need for re-assessing and updating public health definitions.

**Supplementary Information:**

The online version contains supplementary material available at 10.1186/s13690-023-01091-6.

## Background

What is public health? It is a challenging concept that perhaps no single definition will satisfy everyone [1–5]. A national telephone survey of 1234 registered voters conducted in the United States (US) in 1999 found that over half of the respondents misunderstood the term public health [6]. The efforts to define the term public health can be traced back to a century ago to Charles-Edward A. Winslow’s definition which later Sir Donald Acheson in 1988 built on it and put forward his definition as “the art and science of preventing disease, prolonging life and promoting health through the organized efforts of society” [7, 8]. Winslow’s definition is considered one of the most commonly cited definitions of public health [9]. It is still considered valid today [2]. Winslow’s influence can still be seen in many contemporary definitions of public health [1]. In 2011, a concept paper of the World Health Organization (WHO) European Region which investigated several definitions of public health from a selection of key stakeholders concluded that Acheson’s definition can be a useful point of departure [8, 10].

Several studies insisted on the important role of public health definition [9, 10]. According to the Pan American Health Organization (PAHO), [9]. the definition of public health strengthens public health. “Operationalising activities, intelligence, systems, skills and competencies for public health is consequent on the definition of public health” [10]. A clear definition of public health helps people who work in, are served by, or study the system to sort out its components, understand it, and work to improve it [4].

During the last hundred years, several major epidemics and pandemics have afflicted humanity. Currently, the world has been confronting Severe Acute Respiratory Syndrome Coronavirus-2 (SARS-CoV-2) causing Coronavirus disease 2019 (COVID-19). Since December 2019 when the first cases of COVID-19 have emerged in Wuhan, China, this novel infectious disease has spread to millions of people worldwide [11]. The outbreak of COVID-19 was characterized as a pandemic by WHO on 11 March 2020 [11]. In addition to these major public health issues, there are new phenomena such as atmospheric warming, sea level rise, mountain glacier loss, and ocean acidification [12]. These phenomena lead to extreme weather events such as storms, floods, droughts, heatwaves, fires, and many more, which threaten the lives of billions of people around the world [12]. Whether these new phenomena can be considered and called public health issues depends on the definition of public health [1]. Needless to say, by considering a phenomenon as a public health issue, attention is drawn to the fact that such an issue is common or increasing within a population [1]. Calling a phenomenon public health issue could highlight the fact that such an issue does not solely depend on individual actions but is influenced by other conditions including socio-economic situations [1]. When a phenomenon is considered a public health issue, it might trigger the idea that it should be treated differently, perhaps through collective or governmental rather than individual action [1]. Prioritisation and treatment with a particular urgency could be expected when a phenomenon is labelled as a public health issue [1]. These consequences are not exclusive and many more can be expected [1].

The concept of public health is not fixed and has been changing over time [9, 13]. What is included in public health has been evolving in accordance with our understanding of reality and the instruments available for intervention [9, 5]. Due to the complexity of public health in today’s world, it is a multifaceted concept in constant flux [9]. “All the different facets of this concept deserve to be examined carefully from all possible angles, as they manifest themselves through the many different ways in which they are defined and acted on” [9]. The ever-changing nature of public health requires a continuous need for reassessing and updating its definition [14]. Recently, Nutbeam and Muscat talked about Health Promotion Glossary 2021 which is the first full review and revision of the first Health Promotion Glossary commissioned by the WHO in 1986, fully revised in 1998 [15–17]. “This revision provides an updated overview of the many ideas and concepts which are central to contemporary health promotion” [15]. One of the definitions modified in this revision was public health [15]. However, this modified definition is taken from the Dictionary of Public Health published in 2007 [18].

This background leads us to a consideration of whether current definitions stemming from long-standing definitions can respond to today’s public health challenges. Before taking this point into consideration, we needed to identify and review available public health definitions, which was the objective of the current study. To achieve this objective, a scoping review of the literature was conducted, which is discussed in detail in the next section.

## Methods

### Search strategy and selection criteria

A scoping review of peer-reviewed and grey literature was conducted to identify available public health definitions. This review was prepared according to the framework of the Preferred Reporting Items for Systematic Review and Meta-analysis (PRISMA) guidelines [19].

In order to gain familiarity with the previous studies and aid with the identification of key concepts and words, several preliminary searches were conducted. Then, four databases including PubMed (from its inception until June 06, 2022), Web of Science (from its inception until June 06, 2022), EBSCOhost (from its inception until June 06, 2022), and Embase (from its inception until June 08, 2022) were searched to identify relevant literature. Grey literature was searched using Google Scholar (from its inception until June 06, 2022) and the first 100 search results sorted by relevance were compared against the inclusion criteria. Moreover, reference lists of publications included in the scoping review were screened manually for additional relevant publications. No language restriction was applied at this stage.

The search for the relevant literature was conducted using the following keywords in the title and abstract of the literature: “public”, “health”, “definition”, and “meaning”. The final search results were exported into an Excel spreadsheet, and duplicates were removed.

Publications were included if they satisfied all of the following eligibility criteria: (1) All types of scientific publications such as articles, editorials, viewpoints, guidelines, etc.; (2) English-language publications providing an original definition of public health.

**Fig. 1:**
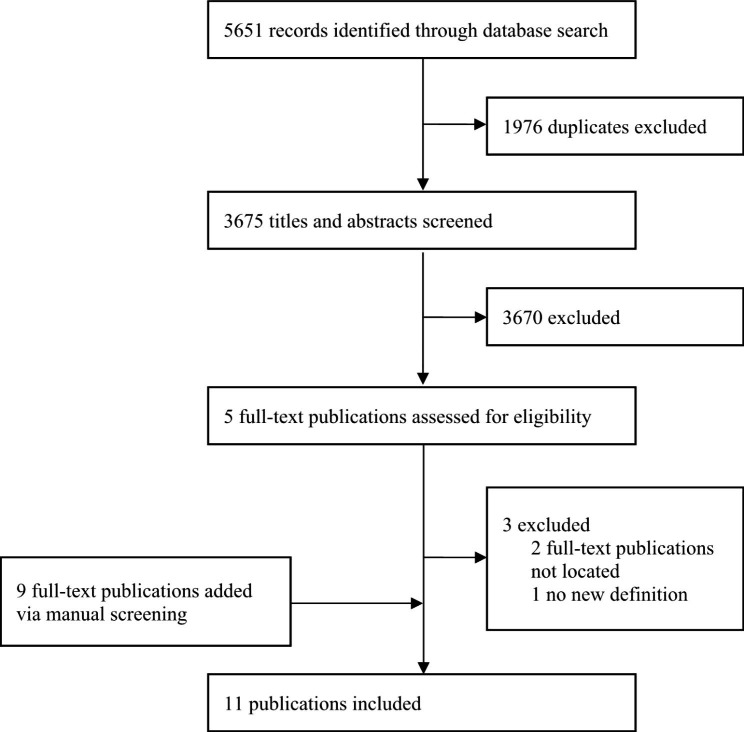
Flow diagram of the scoping review process (from inception until June 06, 2022) based on the Preferred Reporting Items for Systematic Review and Meta-analysis (PRISMA) [19]

The selection of relevant publications was conducted in three stages: (1) Screening of the title and abstract conducted by the first author (RA); (2) Full text screening completed independently by the first and second authors (RA and BB). Raised discrepancies resolved through discussion until consensus was reached; and (3) data extraction and collation. These stages were summarized in the Preferred Reporting Items for Systematic Review and Meta-analysis (PRISMA) flow diagram (see Fig. 1).

Figure 1: Flow diagram of the scoping review process (from inception until June 06, 2022) based on the Preferred Reporting Items for Systematic Review and Meta-analysis (PRISMA) [19]

### Data analysis

Eligible publications were reviewed by RA and BB independently and the following data were extracted: Title, author(s), year of publication, country/region, type of publication, disciplinary fields of publications, aims and objectives of the publication, and public health definitions (see Table 1). Discrepancies were resolved through discussion until a consensus was reached.

**Table 1:** Characteristics of 11 studies included in the scoping review from inception until June 06, 2022

Num	Author(s)/Study Name, Year	Country/ Region	Publication type	Disciplinary fields of publications	Objectives of publication	Definition
1	Winslow, (1920) [7]	North America	Original research articles	Health and public health	Formulating scopes and tendencies of modern public health.	“Public health is the science and the art of preventing disease, prolonging life and promoting physical health and efficiency through organised community efforts for the sanitation of the environment, the control of community infections, the education of the individual in principles of personal hygiene, the organisation of medical and nursing service for the early diagnosis and preventative treatment of disease, and the development of social machinery which will ensure to every individual in the community a standard of living adequate for the maintenance of health.”
2	Acheson, (1988) [8]	Europe	Book	Health and public health	Reviewing the future development of the public health function	“the art and science of preventing disease, prolonging life and promoting health through the organized efforts of society.”
3	Institute of Medicine, (1988) [4]	North America	Book	Health and public health	Analysing the public health situation in the United States (US) and presenting a plan of action that provides a solid foundation for a strong public health capability throughout the nation.	“Public health is what we, as a society, do collectively to assure the conditions in which people can be healthy.”
4	WHO, (1998) [24]	Europe	Report	Health and public health	Providing expert assessments of the global health situation and projecting health trends to the year 2025.	“The art of applying science in the context of politics so as to reduce inequalities in health while ensuring the best health for the greatest number.”
5	WHO Regional Office for Europe, (1999) [25]	Europe	Book	Health and public health	Providing new health for all policy framework for the WHO European Region	“The science and art of preventing disease, prolonging life and promoting mental and physical health and efficiency through organized community efforts. Public health may be considered as the structures and processes by which the health of populations is understood, safeguarded and promoted through the organized efforts of society.”
6	Forsetlund and Bjorndal, (2001) [26]	Europe	Original research articles	Public health	Investigating “whether there is a potential for greater use of research-based information in public health practice in a local setting.”	“Broadly speaking, the practice of public health may be defined as the organisation and analysis of medical knowledge in such a way that it may be utilised by society in the making of decisions in health related questions.”
7	Gostin, (2001) [27]	North America	Original research articles	Public health, medicine, law, ethics, and human rights	Providing a greater understanding of the related fields of public health, ethics, and human rights.	“Society’s obligation to assure the conditions for people’s health.”
8	Childress and colleagues, (2002) [28]	North America	Original research articles	Public health, medicine, law, and ethics	Providing a rough conceptual map of the terrain of public health ethics.	“Public health is primarily concerned with the health of the entire population, rather than the health of individuals. Its features include an emphasis on the promotion of health and the prevention of disease and disability; the collection and use of epidemiological data, population surveillance, and other forms of empirical quantitative assessment; a recognition of the multidimensional nature of the determinants of health; and a focus on the complex interactions of many factors - biological, behavioral, social, and environmental - in developing effective interventions.”
9	Pan American Health Organization, (2002) [9]	Americas	Book	Health and public health	Reflecting on the context (Americas) in which public health is perceived and practiced and setting out the extent to which those functions that are essential to promoting and preserving the public’s health are being discharged.	“Public health is an organized effort by society, primarily through its public institutions, to improve, promote, protect and restore the health of the population through collective action.”
10	Rothstein, (2002) [14]	North America	Original research articles	Law, medicine, and bioethics	Providing a clear and narrow definition of public health.	“The term “public health” is a legal term of art, and it refers to specifically delineated powers, duties, rights, and responsibilities. Even beyond its legal usage, public health applies to specific institutions and individuals, such as public health departments and public health officials.”
11	Heller and colleagues, (2003) [23]	Europe	Original research articles	Health and public health	Providing a new and wide definition of public health that recognizes the centrality of the public and meets the expectations of those who work in the discipline and the public to whom they are accountable.	“Use of theory, experience and evidence derived through the population sciences to improve the health of the population, in a way that best meets the implicit and explicit needs of the community (the public).”

## Results

The electronic searches conducted in the above-mentioned databases identified a total of 5651 publications, resulting in 3675 unique publications to be screened for inclusion following the removal of duplicates (see Fig. 1). Based on the eligibility criteria, the titles and abstracts were assessed for their relevance, resulting in five publications being retained. Of these, two publications were excluded as their abstracts and full texts were not available [20, 21]. The full texts of the remaining publications, three publications, were obtained and after applying the eligibility criteria, one publication was excluded as it did not introduce a new definition of public health [22]. Therefore, two publications were included [14, 23]. An additional nine publications were identified through the manual screening [4, 7–9, 24–28]. A total 11 of publications were included in the scoping review (see Fig. 1). Characteristics of the included publications are shown in Table 1.

Of the 11 publications included in this review, six publications were original research articles published in 1920, 2001, 2002, and 2003 [7, 26–28, 14, 23]. Four publications were books published in 1988, 1999, and 2002 [8, 4, 25, 9]. One publication was a report published in 1998 [24]. All the publications were authored in North America and Europe mainly. Disciplinary fields of the publications included health, public health, medicine, law, ethics, bioethics, and human rights (see Table 1).

Of the 11 publications included in this review, only two publications set providing a new definition of public health as the aim and objective of their studies [14, 23]. Formulating scopes and tendencies of modern public health, reviewing the future development of the public health function, analysing the public health situation in the US and presenting an action plan for a strong public health capability, providing expert assessments of the global health situation and projecting health trends, providing new health for all policy framework, investigating the use of research-based information in public health practice, providing a greater understanding of the related fields of public health, ethics, and human rights, providing a conceptual map of the terrain of public health ethics, and reflecting on public health in the context of Americas were stated as the aims and objectives of the other publications (see Table 1).

All the publications under review provided explicit definitions. All the definitions which are provided in Table 1 were formulated by their authors without any references to other definitions.

The publications under review reported several rationales for providing a new definition of public health. Winslow sought to provide a more inclusive definition [7]. “If the foregoing outline of the problems of public health be accepted as correct, it will be obvious that the field as thus visualized is no small and restricted one” [7] …. “If we are looking to the future we must conceive our subject in terms no smaller than those of the [provided] definition” [7]. Acheson looked for a wide definition of the term public health [8]. In the past, public health has been rather narrowly interpreted and associated with sanitary hygiene and epidemic disease control [8]. He preferred a broader definition which gives “as much weight to the importance of lifestyle as to environmental hygiene in the preservation and promotion of health” [8]. Institute of Medicine believed that understanding of public health should not be restricted to what health departments do [4]. A clear definition helps those who work in, are served by, or study the system to understand it and work to improve its performance [4]. The need for expanding and clarifying the meaning of public health based on public health challenges was another rationale stated by PAHO [9]. Traditional cornerstones of public health such as prevention and control of communicable diseases or environmental sanitation continue to be important; however, current definitions of public health should include much more [9]. Moreover, defining public health in terms of what the government does is no longer sufficient [9]. “Government should in fact play a central and fundamental role in public health today. However, not everything that government does in terms of health can be regarded as public health, just as public health cannot remain limited to government action” [9]. Rothstein sought to narrow the scope of public health definition and opposed using “the term “public health” as an open-ended descriptor of widely divergent efforts to improve the human condition” [14]. There is an ongoing need to reassess scientific, ethical, legal, and social underpinnings of public health as it evolves [15]. However, considering so many activities as public health just because they interfere with the health of individuals and populations does not mean that eliminating them is part of the mission of public health and can solve the problem of poor health [14]. Broad definition of public health will not eliminate forms of human privations to call them public health issues [14]. Heller and colleagues’ rationale was to offer a broader and more inclusive definition which helps public health professionals interpret their own roles [23]. They tried to provide a wider definition of public health by recognizing the centrality of the public [23]. “The practice of public health has been criticized as being too involved with a narrow, managerial agenda focused on health care rather than the wider horizons of public good” [23]. Moreover, they attempted to produce a clear definition that “meets the expectations of those who work in the discipline and the public to whom they are accountable” [23].

## Discussion

This scoping review was conducted to identify available public health definitions. While previous studies have emphasized the importance of re-assessing and updating definitions of public health, [1, 10] the results of the present study indicated a noticeable lack of updated definitions. Of the 11 definitions included in this review, the latest original definitions date back to about 20 years ago. During the last two decades, the world has witnessed the emergence and reemergence of viral outbreaks of Severe Acute Respiratory Syndrome Coronavirus (SARS-CoV) in 2002, Influenza A virus subtype H1N1 (A/H1N1) in 2009, Middle East Respiratory Syndrome Coronavirus (MERS-CoV) in 2012, Ebola virus in 2013, and the SARS-CoV-2 in 2019 [29, 30]. Findings of the study led us to a consideration of whether current definitions can fully respond to today’s public health challenges. The situation could be more challenging as public health is a “concept with shifting parameters and multiple interpretations” [10]. Scholars have insisted on the fact that public health is in constant flux [1, 9, 13]. Considering the findings of the study and the ever-changing nature of public health issues, it can be argued that there is an urgent need for re-assessing and updating public health definitions.

In the last twenty years, new phenomena, such as global warming and climate change, affect the health and well-being of billions of people around the world. Considering these phenomena as public health issues could lead to increased attention, prioritization, different treatment, and many more actions [1]. Whether these new phenomena can be considered and called public health issues depends on the definition of public health [1]. Therefore, it seems that updating public health definitions is needed.

The results indicated that the definition of public health plays a crucial role in reaching objectives such as analysing public health situations locally and globally, providing policies and action plans, and many more. This result was in accordance with what previous studies insisted on, which is the important role of public health definition in understanding, shaping, and strengthening public health [4, 9]. It can be argued that updating public health definition is needed as an outdated definition might not be able to fulfil such an important role.

According to the findings of the study, a “narrow–broad distinction” [1] can be drawn between the identified public health definitions. One group of the definitions was provided to present a narrow approach, while the other group was provided to present a broad one. Narrow definitions focus more on health factors such as sanitation, infectious disease control, screening programmes, or health education [1]. Broad definitions deal with all of the factors that might affect health, including societal, cultural, and economic determinants of health [1, 14, 31]. Rickles called this distinction “local’ and ‘nonlocal’ since they concern factors that act directly on individuals in the former case and more indirectly in the latter case” [5].

According to the findings of the study, eight out of the 11 publications included in this review were authored in higher-income countries. In other words, the majority of available public health definitions were authored in higher-income countries, which may unevenly illustrate the interests and priorities of stakeholders from higher-income countries. The findings suggest a need for greater diversity and inclusion in providing definitions of public health.

Health, medicine, law, ethics, bioethics, and human rights were the disciplinary fields of the publications included in this review, which could be due to the fact that public health is a massively interdisciplinary field, incorporating epidemiology, biology, sociology, economics, psychology, and more [5].

The present study has the strength of being the first scoping review conducted to identify available public health definitions. However, the limitations of our study need to be considered. One limitation of our study is that only publications focused on definition of public health were included, which might lead to the absence of studies which provided a new definition of public health without focusing on the subject matter exclusively. Manual screening of reference lists of publications included in the scoping review was used to add relevant publications that had not been initially identified through database searching. This ensured that the review was exhaustive. However, it means that some conclusions may have been influenced by this manual search strategy. Despite not restricting the language of publication, only English keywords were searched, which could lead to the exclusion of non-English publications providing public health definitions. Another limitation is that only publications written in English were included. Future reviews should include non-English studies to have a better understanding of the situation.

## Conclusions

Most frequently cited definitions of public health stemmed from long-standing definitions. Despite the emphasis on the importance of re-assessing and updating definitions of public health, there is a noticeable lack of updated definitions. This lack raises several concerns including responding to today’s public health challenges. Considering previous studies, the findings of this study, and the ever-changing nature of public health issues, there is an urgent need for re-assessing and updating public health definitions. Future studies could focus on providing new definitions that fit the present global society.

## Electronic supplementary material

Below is the link to the electronic supplementary material.

Supplementary Material 1

## Data Availability

All data generated or analysed during this study are included in this published article.

## List of Abbreviations

US: The United States
WHO: The World Health Organization
PAHO: The Pan American Health Organization
COVID-19: Coronavirus disease 2019
