# Supplementary material for: What is public health? a scoping review
Source: Arch Public Health. 2023 May 10;81:86. doi: 10.1186/s13690-023-01091-6 (PMC10170773; doi:10.1186/s13690-023-01091-6)
Supplement: Supplementary file 1 — Supplementary Material 1 [file 13690_2023_1091_MOESM1_ESM.docx]

**What is public health? a scoping review**

**What is known**

The definition of the term “public health” plays an influential role in public health. It strengthens and improves public health. During the last hundred years, several major epidemics and pandemics have afflicted humanity. In addition to these major public health issues, there are new phenomena, such as global warming and climate change, that affect the health and well-being of billions of people around the world. Considering these phenomena as public health issues could lead to several actions, including increased attention, prioritization, different treatment, and many more. Whether these new phenomena can be considered and called public health issues depends on the definition of public health.

Moreover, public health is not a fixed concept and has been changing over time. A continuous need for re-assessing and updating public health definitions is required due to its ever-changing nature. However, most frequently cited definitions of public health have stemmed from long-standing definitions, which raises several concerns, including whether these definitions can respond to today’s public health challenges. The present study aimed to identify and review available public health definitions in the first place. Therefore, several preliminary searches were conducted. We searched PubMed, Web of Science, and EBSCOhost without language restrictions using the keywords “public”, “health”, “definition”, and “meaning” from database inception until 10 May 2022, for publications that focused on definitions of public health. We identified one rapid review that examined a few definitions of public health, but it did not provide an original definition. We did not identify any other scoping or systematic reviews that focused on the subject matter.

**What does the study adds**

To our knowledge, this study is the first scoping review to identify available public health definitions. This study revealed that definitions of public health have not been re-assessed and updated in the last twenty years, despite recent major epidemics and pandemics, the emergence of new phenomena, and the ever-changing nature of public health issues. This scoping review showed that there is an urgent need for re-assessing and updating public health definitions.

The study revealed a need for greater diversity and inclusion in providing definitions of public health as the majority of available definitions were authored in higher-income countries, which may unevenly illustrate the interests and priorities of stakeholders from higher-income countries.

In accordance with previous studies, this scoping review emphasised the fact that public health is a massively interdisciplinary field, incorporating epidemiology, biology, sociology, economics, psychology, etc.

**What the implications are for clinical practice, public health and/or research**

The absence of updated definitions of public health revealed by this scoping review calls for a definition that fits the present global society. Such a definition is crucial for reaching objectives such as analysing public health situations locally and globally, providing policies and action plans, understanding, shaping, and strengthening public health, and many more. The present review showed the gap that needs to be filled for public health to serve and fulfil its tasks. This study could be a starting point for further studies focusing on providing new definitions.
